# Burnout among doctors in China through 2018: A protocol of systematic review and meta-analysis

**DOI:** 10.1097/MD.0000000000017117

**Published:** 2019-09-13

**Authors:** Qin Zheng, Kun Yang, Xue Wang, Zhenghang Ou, Xiaopeng Su, Jing Zhang, Miao Qu

**Affiliations:** a Department of Neurology, Third Affiliated Hospital, Beijing University of Chinese Medicine; b Department of Evidence-Based Medicine; c Department of Library; d Department of Neurology, Xuanwu Hospital, Capital Medical University, Beijing, China.

**Keywords:** burnout, Chinese doctor, prevalence, protocol, systematic review

## Abstract

**Background:**

Burnout negatively impacts the mental and physical health doctors. More seriously, it leads to poor patient care. In China, the situation is severe and more efforts are needed to reveal the epidemiological characteristics of doctor burnout to develop improved strategies of alleviating it. Due to the large number of heterogeneous and sample size-restricted surveys currently published, meta-analysis and systematic reviews are critical to a thorough understanding of burnout among Chinese doctors.

**Methods:**

The Cochrane Collaboration criteria and the Preferred Reporting Items for Systematic Reviews and Meta-Analyses (PRISMA) will be followed to conduct and report the systematic review. We will conduct a comprehensive search on the data bases of China National Knowledge Infrastructure (CNKI), WanFang, PubMed, EMBASE, PsycINFO, and the Cochrane Library from their inception to December 2018. Prospective cohort and cross-sectional studies that described the prevalence of Chinese doctors’ prevalence will be eligible for inclusion. The risk of bias and methodological quality of the included studies will be assessed using a risk of bias tool and the Cochrane guidelines for observational studies. A generalized linear mixed model framework with the Poisson likelihood and the log link function will be used to access the incidence rate ratio. Multivariate Poisson regression framework will be conducted to adjust modeling heterogeneity and confounders, like difference regions and time periods. The risk of bias, heterogeneity, and quality of evidence will be assessed in accordance with the aforementioned guidelines.

**Results:**

The primary outcome will be the prevalence and distribution of 3 dimension of burnout in Chinese doctors, and the second will be the difference of prevalence between difference regions and time periods.

**Discussion:**

This systematic review and meta-analysis will help us to reveal the prevalence, characteristics, timeline, and correlation between these factors in burnout; we expect our work may provide a scientific basis for further prevention and intervention of burnout in Chinese doctors, eventually to improve the quality of health care.

**PROSPERO registration number:**

CRD42018104249.

## 1 Introduction

Burnout is a syndrome resulting from overload and stress during work, and the prevalence is increasingly in human service occupations, especially among professional health practitioners who deal with persons.^[1–4]^ Maslach et al^[1]^ defined burnout as a prolonged response to chronic emotional and interpersonal stressors due to work, which is characterized by 3 dimensions of constructs, namely, emotional exhaustion, depersonalization, and reduced personal accomplishment. Burnout not only negatively affects well-being of health care practitioners, but also results in suboptimal patient care.^[5,6]^ In America, 54.4% physicians had at least 1 symptom of burnout, and in New Zealand, the job burnout prevalence of doctors was as high as 50%.^[7,8]^

In China, for every 1000 people, only 1.2 doctors to provide health care services, compared with 2.5 and 3.9 in the United States and Germany, respectively.^[9]^ During the past 3 decades, Chinese economy has developed rapidly; meanwhile, increasing health demand driven by the improving economy has led to an excessive burden on Chinese doctors.^[10,11]^ A previous study indicated that among Chinese doctors, one-third have experienced conflict with patients, there are high incidence rates of depressive symptoms and suicide attempts,^[12]^ all of which might be induced by burnout. Therefore, it is critical to investigate and comprehend the status of burnout among Chinese doctors to establish strategies for reducing burnout and improving the quality of health care services.

Although a considerable number of investigations have been conducted and published, most data derived merely from regional annual surveys, particular subtypes of doctors (neurologist, anesthesiologist, etc.), or limited samples,^[10,13–15]^ these studies were heterogeneous and only investigated the short term, failing to represent the overall situation and long-term trend in burnout in the entire population of Chinese doctors. Thus, a meta-analysis and systematic review is essential for thoroughly comprehending burnout among Chinese doctors.

To the best of our knowledge, systematic review and meta-analysis based on investigations of burnout in Chinese doctors are still rare. Only 1 publication was found, but it included only 11 studies published in English in the systematic review, and no meta-analysis was conducted.^[16]^ Therefore, it is critical and essential to conduct a systematic review and meta-analysis of burnout in Chinese doctors based on studies published both in English and Chinese, revealing the characteristics, timeline, risk factors, and correlation between factors with regard to occurrence of burnout. Thus, this study may serve as a solid foundation of establishing countermeasures and providing suggestions for reducing burnout, eventually improving health care setting and quality of health care provided by doctors.

## 2 Methods

### 2.1 Protocol registration

The protocol has been registered on the PROSPERO website as CRD42018104249 (http://www.crd.york.ac.uk/PROSPERO/display_record.php? ID = CRD42018104249).

### 2.2 Criteria for included and excluded studies

We will conduct a comprehensive search of China National Knowledge Infrastructure (CNKI), WanFang, PubMed, EMBASE, PsycINFO, and the Cochrane Library databases from their inception to December 2018.

The MeSH Terms and related keywords are as follows: “China”\China\Chinese\“Burnout, Professional”\burnout\“Physicians”\physician\doctor\doctors\“Medical-Staff”\medical staffs\“Allergists”\allergist\“Anesthesiologists”\anesthesiologist\“Cardiologists”\cardiologist\“Dermatologists”\dermatologist\“Endocrinologists”\endocrinologist\“Gastroenterologists”\ga-stroenterologist\“General Practitioners”\general practitioner\“Geriatricians”\geriatrician\“Hospitalists”\hospitalist\“Nephrologists”\nephrologist\“Neurologists”\neurologist\“Oncologists”\oncologist\“Ophthalmologists”\ophthalmologist\“Osteopathic Physicians”\“Otolaryngologists”\otolaryngologist\“Pathologists”\pathologist\“Pediatricians”\pediatrician\“Neonatologists”\neonatologist\“Physiatrists”\physiatrist\“Pulmonologists”\pulmonologist\“Radiologists”\radiologist\“Rheumatologists”\rheumatologist\“Surgeons”\surgeon\“Neurosurgeons”\neurosurgeon\“Urologists”\urologist\obstentrician\gynecologist\orthopedist\“Dentists”\dentist\.

The inclusion criteria are as follows: studies with participants who are Chinese doctors; prospective studies; cross-sectional studies; publications in English and Chinese; studies which used measurement tool of Maslach Burnout Inventory (MBI)\Maslach Burnout Inventory-General Survey (MBI-GS)\Maslach Burnout Inventory-Human Services Survey (MBI-HSS)\Chinese Maslach Burnout Inventory (CMBI)\the Chinese version of Copenhagen Burnout Inventory (C-CBI)\Burnout Inventory-Physician Survey (BI-PS); and studies that provide the data necessary to calculate prevalence.

The exclusion criteria are as follows: studies that have medical technicians and paramedics as subjects; duplicate publications; retrospective studies and interventional studies; and studies from which the data needed for the meta-analysis could not be obtained.

### 2.3 Outcome

The primary outcomes will be the total prevalence of burnout, the specific prevalence of burnout in different regions in China, and the timeline. The secondary outcomes will be comparisons of burnout scores among doctors of different genders, marital status, and professional titles.

### 2.4 Study selection and data extraction

**Figure 1:**
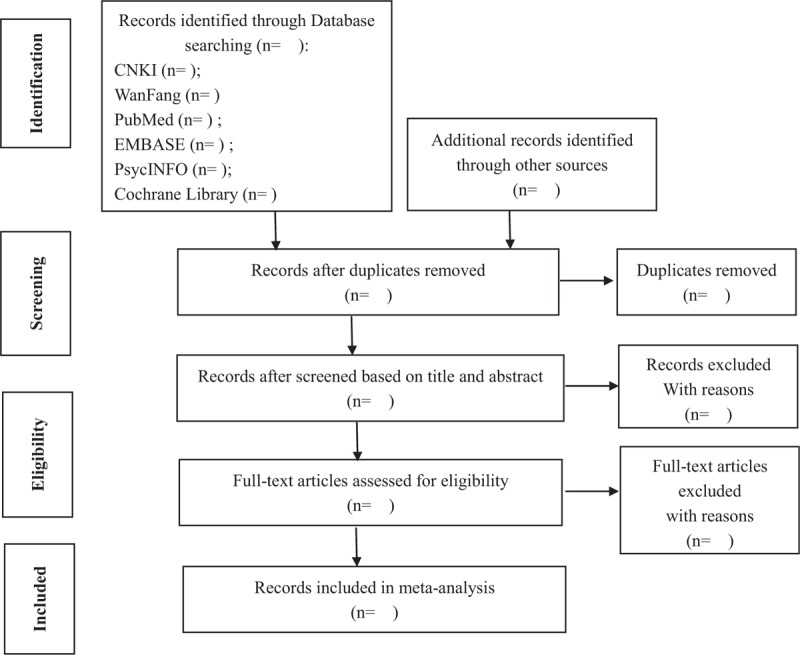
Flow diagram of article selection. Identification: we will identify records through database searching and other sources. Screening: we will remove duplicates and exclude records those are not fit our inclusions after screening title and abstract. Eligibility: we will assess full-text articles for eligibility. Included: We will include records that are screened by inclusion criteria and exclusion criteria for meta-analysis. CNKI = China National Knowledge Infrastructure.

Two authors will independently screen the titles or abstracts or full texts of all eligible studies. Disagreements between 2 authors will be resolved by consensus. We will present the process of search and study selection using a flow process chart (Fig. 1). Two authors will use the inclusion criteria and exclusion criteria to independently extract literatures, screen the titles and abstracts of all eligible studies, and then independently review full-text articles of studies that meet the criteria. The following information will be extracted with a standard form: title, first author name, year of publication, study region, study time, sample size, mean age, gender, measurement tool, and results. Detail of consulting a third author or the original authors will be contacted for further information if necessary.

### 2.5 Risk of bias assessment and quality of study

Because all studies are observational studies, the quality of the included studies will be evaluated with reference to the “quality evaluation standard for observational studies” proposed by Hoy et al.^[17]^ The scale is composed of 11 items. The answer “yes” is 1 point, and “no” or “not clear” is not score. Quality scores will be presented in a table.

### 2.6 Statistical analysis

Heterogeneity across studies will be tested by using the *I*^2^ statistic,^[18]^ which is a quantitative measure of inconsistency across studies. Studies with an *I*^2^ statistic of 25% to 50%, 50% to 75%, and >75% will be considered as low, moderate, and high heterogeneity, respectively. Fixed or random effects models will be used appropriately according to the *I*^2^ statistic. The pooled prevalence of the outcome variable will be expressed as overall rate with 95% confidence intervals and the model will be set based on a Poisson regression framework. The inverse of the squared root of the study sample size will be used to down weigh the large studies. The clustering of data points within each study will be taken into consideration as well.

Overall summaries of the meta-estimates (and confidence intervals) will be reported, all of them are expressed as percentages. In addition, we will perform a sensitivity analysis to explore possible explanations for heterogeneity and to examine the influence of various exclusion criteria on the overall estimate. A generalized linear mixed model framework with the Poisson likelihood and the log link function will be used to access the incidence rate ratio. Multivariate Poisson regression framework will be conducted to adjust modeling heterogeneity and other confounders, such as study design (randomized controlled trials [RCTs] versus non-RCTs), mean age, and gender.^[19]^

We will assess the robustness of our conclusions and investigate the influence of a single study on the overall pooled estimate by omitting 1 in each turn. Meta-regression analysis will be performed to explore the time trend of publication year. Potential publication bias will be assessed by visually inspecting the funnel plots in which the estimates will be plotted against their standard errors. The presence of publication bias will also be evaluated using the Egger and Begg tests. *P* < .05 is considered to be statistically significant. All analyses will be performed on Stata Statistical Software (version 14.0, Stata Corp, College Station, TX).

### 2.7 Reporting of this review

The systematic review and meta-analysis will be reported following the guideline of Preferred Reporting Item for Systematic review and Meta-Analyses (PRISMA) and guideline of Meta-Analyses of observational studies.^[20,21]^

## 3 Discussion

In summary, this systematic review and meta-analysis will help us to reveal the prevalence, characteristics, timeline, and correlation between these factors in burnout; we expect our work may provide a scientific basis for further prevention and intervention of burnout in Chinese doctors, eventually to improve the quality of health care.

## Author contributions

**Conceptualization:** Miao Qu.

**Data curation:** Zhenghang Ou, Xiaopeng Su.

**Methodology:** Kun Yang, Xue Wang.

**Writing – original draft:** Qin Zheng.

**Writing – review & editing:** Jing Zhang, Miao Qu.
